# Application of the BOPPPS-CBL teaching model in gynaecological clinical internships: a randomized controlled trial

**DOI:** 10.3389/fmed.2026.1796370

**Published:** 2026-05-08

**Authors:** Lin Ling, Wenyan Wang, Juanjuan Fu, Xue Wang, Yu Wang, Jing Wang

**Affiliations:** 1 Department of Obstetrics and Gynecology, The Second Affiliated Hospital of Anhui Medical University, Hefei, China; 2 Department of Ophthalmology, The Second Affiliated Hospital of Anhui Medical University, Hefei, China

**Keywords:** BOPPPS, case-based learning, clinical internships, gynecology, medical education

## Abstract

**Background:**

Clinical internships are pivotal for undergraduate medical students transitioning from theoretical learning to clinical practice. However, traditional lecture-based learning (LBL) in gynaecological clinical internships often limits active engagement and the development of clinical competence. This study aimed to evaluate the educational value of integrating Case-Based Learning (CBL) with the BOPPPS model (Bridge-in, Objective, Pre-assessment, Participatory Learning, Post-assessment, Summary) in gynaecological internship teaching, with a focus on its impact on interns’ learning outcomes and learning experience.

**Objectives:**

(1) To compare interns’ theoretical knowledge mastery between BOPPPS-CBL and traditional LBL groups; (2) To evaluate the BOPPPS-CBL model’s impact on interns’ clinical reasoning, practical skills, and doctor-patient communication; (3) To investigate differences in learning experience, satisfaction, and pressure between the two teaching modes; (4) To provide an effective, innovative teaching scheme for gynaecological clinical internships.

**Methods:**

A total of 90 undergraduate medical students who completed gynaecological clinical internships at the Second Affiliated Hospital of Anhui Medical University between July and December 2025 were randomly allocated into two groups using a random number table: the Lecture-Based Learning group (LBL group, *n* = 45) and the BOPPPS-CBL group (*n* = 45). Teaching effectiveness and students’ learning attitudes were comprehensively assessed using pre-class tests, post-class tests, Mini-Clinical Evaluation Exercise (Mini-CEX), direct observation of procedural skills (DOPS), and questionnaire surveys.

**Results:**

The results demonstrated that theoretical examination scores and Mini-CEX indicators assessing clinical reasoning in the experimental group were significantly higher than those in the control group (*p* < 0.001). For DOPS indicators evaluating clinical skills, no statistically significant difference was observed in the informed consent component (*p* = 0.458), whereas all other components showed statistically significant differences between groups (*p* < 0.05). Questionnaire findings indicated that both teaching quality and students’ self-evaluated learning outcomes were significantly higher in the BOPPPS-CBL group than in the LBL group (all *p* < 0.001). However, students in the BOPPPS-CBL group also reported significantly increased learning pressure (all *p* < 0.001).

**Conclusion:**

The integration of the BOPPPS model with CBL significantly enhances gynaecological interns’ theoretical knowledge, comprehensive clinical competence, and practical skill proficiency, while improving learning experience and satisfaction. BOPPPS-CBL represents an effective and innovative teaching approach for gynaecological clinical internships.

## Introduction

Clinical internships is a critical stage for undergraduate medical students to translate theoretical knowledge into clinical competence and to develop their initial clinical reasoning skills (1). As a discipline that emphasises both theoretical understanding and practical application, gynaecology places particular importance on clinical internships training. However, the traditional gynaecological teaching model, which is largely based on listening to lectures and observing instructors’ clinical procedures, presents several limitations. First, students often lack a perceptual understanding of diseases, which hinders their comprehension of theoretical concepts (2). Second, clinical instructors are frequently preoccupied with clinical duties, resulting in limited teacher–student interaction and reduced learning efficiency (3). In addition, owing to the privacy-sensitive nature of gynaecological conditions, interns frequently encounter restrictions when observing patient management or participating in gynaecological examinations (4). Consequently, the internships process becomes monotonous and passive, leading to reduced learning motivation and ultimately constraining the development of clinical skills, clinical thinking, and doctor–patient communication abilities (5). Current consensus indicates that teacher-centred traditional teaching is far less effective than student-centred active learning, which is more conducive to the development of clinical competence and professional growth (6, 7). Therefore, reforming traditional teaching methods has become an urgent priority in gynaecological clinical education to cultivate high-quality medical professionals with strong problem-solving abilities.

Case-Based Learning (CBL) is a teaching approach that guides students to analyse clinical problems through real or well-designed representative cases, thereby promoting active learning and fostering critical thinking and analytical skills among undergraduate medical interns (8). However, traditional CBL often lacks a clear instructional structure and may fail to actively engage all learners, which limits its overall teaching effectiveness (9).

The BOPPPS framework, developed in 1984, consists of six stages designed to enhance student engagement: bridge-in, objective, pre-assessment, participatory learning, post-assessment, and summary (10). Compared with traditional teaching models, this student-centred framework significantly improves teaching efficiency by increasing course satisfaction, strengthening teacher–student interaction, and enhancing the development of clinical analytical skills. Consequently, the BOPPPS model demonstrates multiple advantages and promotes greater student participation (11). Increasing evidence indicates that the BOPPPS model, particularly when adapted to blended teaching formats that integrate complementary learning strategies, can improve educational outcomes and academic performance (12–14). As the model continues to evolve in response to changing educational needs, its proven effectiveness and adaptability highlight its potential for broader application.

The BOPPPS-CBL model integrates the strengths of both approaches by combining the structured and participatory features of BOPPPS with the practical, case-oriented focus of CBL. This integrated teaching model has recently shown promising outcomes in medical education, particularly in nursing education and residency training, but has rarely been applied in undergraduate medical internships teaching (10). Therefore, this study aims to investigate the effectiveness of the BOPPPS-CBL model in gynaecological clinical internship teaching, with the objective of addressing the limitations of traditional teaching methods and enhancing interns’ clinical competence.

## Methods

### Study design and participants

This randomized controlled trial was conducted from July 2025 to December 2025 at The Second Affiliated Hospital of Anhui Medical University, a large comprehensive hospital with established medical teaching and research capacity. Participants were fourth-year undergraduate clinical medical students undertaking clinical internships in the Department of Gynecology. Inclusion Criteria: (1) Have completed the theoretical courses of gynaecology and obstetrics and passed the examination; (2) Volunteer to participate in this study and sign the informed consent form; (3) No history of clinical internships in the Department of Gynecology before; (4) Be able to complete the entire teaching process and all evaluation indicators as required. Exclusion Criteria: (1) Students who have participated in other gynecological teaching reform projects; (2) Students who are absent from more than 2 teaching sessions due to illness, leave or other reasons. A total of 90 students provided written informed consent and were randomly allocated to an experimental group (BOPPPS-CBL group, *n* = 45) or a control group (LBL group, *n* = 45). Randomization was performed using the R programming language. A sequence of random integers was generated, and participants were assigned according to the parity of the numbers: odd numbers were allocated to the LBL group, whereas even numbers were allocated to the BOPPPS-CBL group. The randomization list was generated by an independent statistician to ensure impartiality. Written informed consent was obtained from all participants. The study was approved by the Ethics Committee of the Second Affiliated Hospital of Anhui Medical University (SL-YX2026-033).

### Teaching implementation

Using “uterine myoma” as an example, a 2-h teaching session was delivered.

### LBL method

The traditional LBL method delivered content through a two-hour lecture using slide presentations. First, the teacher explained the relevant theoretical knowledge of uterine myoma in accordance with the syllabus requirements. Subsequently, clinical cases were discussed, covering medical history, initial documentation, diagnostic criteria, differential diagnosis, and treatment strategies. Intern physicians were given 15 min for independent thinking and 30 min for answering questions and discussion. Finally, the teacher summarised and reviewed the key knowledge points.

### BOPPPS-CBL method

**Figure 1:**
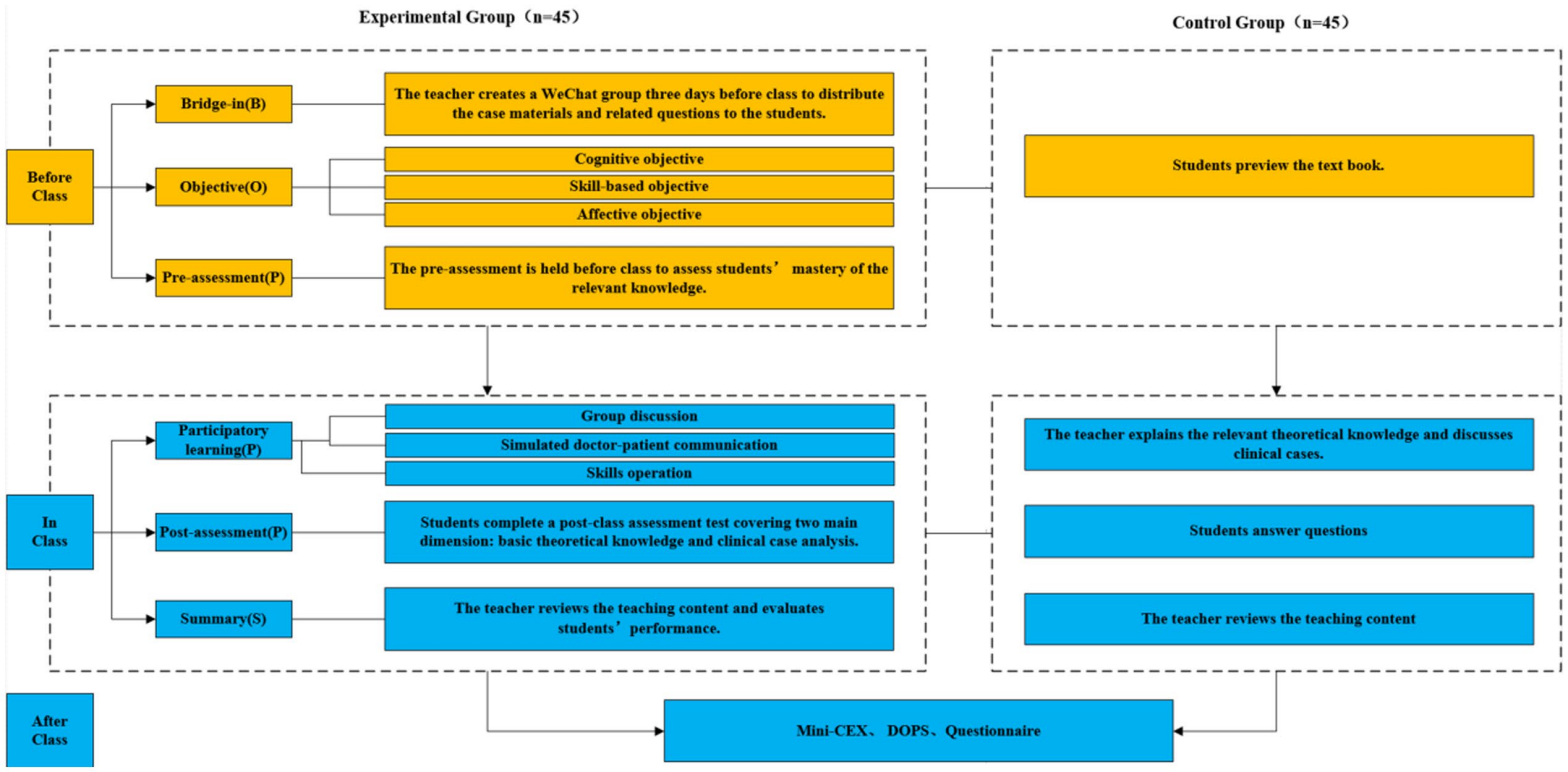
Flowchart of the study design. Exp group, experimental group with the combined BOPPPS and CBL method. Con, control group with the traditional LBL method.

The BOPPPS-CBL model comprised six core components (as illustrated in Figure 1).

1. Bridge-in: A We Chat group was established, and the teacher distributed case materials and related questions to students 3 days prior to the class. For example, a patient consultation video was shared, highlighting the patient’s anxiety arising from heavy menstrual bleeding and repeated failure to conceive, with an ultrasound image of a submucosal myoma presented at the end of the video. Students were guided to reflect on the following questions: “If you were the attending physician for this patient, how would you formulate an initial management plan? Is surgery necessary? How would you balance myoma treatment with her fertility needs?”
2. Objective (5 min): A teaching approach emphasising the ability to describe, operate, and apply knowledge was adopted to improve learning efficiency and to gradually enhance students’ clinical reasoning. Teaching objectives were clearly defined to ensure that they were achievable for students and evaluable for teachers. Three levels of objectives were specified, encompassing cognitive, skill-based, and affective domains.

1. Cognitive objective: Students should master the classification, clinical manifestations, and indications for surgical treatment of uterine myoma.
2. Skill-based objective: Students should be able to independently perform gynaecological examinations and design individualised treatment plans (medication/surgery).
3. Affective objective: Students should demonstrate effective doctor-patient communication and humanistic care in clinical practice, embodying the “love for patients” ethos.

1. Pre-assessment (5 min): A 5-min online test comprising three multiple-choice questions was administered before class, focusing on the clinical symptoms, diagnosis, and treatment of uterine myoma, to assess students’ mastery of the relevant knowledge.
2. Participatory learning (80 min): This constituted the core component of the model, with teaching based on real clinical cases to fully mobilise students’ initiative and to integrate relevant knowledge systematically.
3. Group discussion (40 min): Intern physicians were divided into Group A and Group B. Based on the medical history, ultrasound findings, and blood routine test results provided in the case, students first determined the type of myoma and the degree of anaemia. Subsequently, Group A advocated “hysteroscopic myomectomy followed by natural conception”, whereas Group B proposed “assisted reproduction first, followed by myomectomy combined with caesarean section at delivery”.
4. Simulated doctor-patient communication (20 min): Role-play doctor-patient dialogues were conducted to discuss subsequent treatment options with the patient, including the advantages and disadvantages of myoma surgery and the timing of pregnancy. Any missing content was supplemented by the teacher in a timely manner.
5. Skills operation (20 min): Bimanual examination.
6. Post-assessment (15 min): At the end of the course, students completed a post-class assessment test consisting of five questions covering two main dimensions: basic theoretical knowledge and clinical case analysis. This timely assessment assisted teachers in monitoring teaching effectiveness and optimising lesson plans, while also enabling students to evaluate their mastery of the subject matter.
7. Summary (15 min): The teacher reviewed the teaching content and evaluated student performance. Subsequently, the core principles of the topic were elaborated, key concepts were highlighted, and future advances were outlined. In addition, students were required to construct mind maps or knowledge trees to summarise the knowledge they had acquired.

### Evaluation

The assessment of intern physicians comprised four components: a pre-class test, a post-class test, the Mini Clinical Evaluation Exercise (Mini-CEX), and Direct Observation of Procedural Skills (DOPS). The Mini-CEX included seven dimensions: history taking, physical examination, communication skills, clinical judgement, organization efficiency, professionalism, and overall clinical competence. Each item was scored on a 9-point scale, with scores of 1–3, 4–6, and 7–9 corresponding to “unsatisfactory,” “satisfactory,” and “superior,” respectively (15) DOPS, which assesses technical skills, evaluated items including indications and contraindications, obtaining informed consent, pre-procedural preparation, technical ability, communication skills, humanistic qualities/professionalism, and overall procedural competence, using scoring criteria similar to those of the Mini-CEX (16).

In addition to the theoretical examination, a questionnaire survey was administered to assess students’ learning attitudes and perceived learning outcomes, including self-directed learning enthusiasm, increased study workload, understanding of teaching content, student-teacher interaction, satisfaction with the teaching mode, satisfaction with teaching effectiveness, self-confidence, and interest in learning. Each item was rated using a 5-point Likert scale, with 5 points indicating very satisfied, 4 points satisfied, 3 points neutral, 2 points dissatisfied, and 1 point very dissatisfied. Given the relatively small sample size of this study, scores of 4–5 were combined as “agreement,” a score of 3 was classified as “neutrality,” and scores of 1–2 were combined as “disagreement.”

### Statistical analysis

Data normality was assessed using the Shapiro–Wilk test. Depending on the distribution, results were presented as mean ± standard deviation (SD) or as median values with the interquartile range (IQR). Categorical variables were analysed using the chi-square test. All statistical analyses were performed using SPSS version 23.0 (SPSS Inc., Chicago, USA). All tests were two-tailed, and statistical significance was set at *p* < 0.05.

## Results

### Baseline characteristics of students

A total of 90 fourth-year undergraduate students were enrolled and randomly assigned to two groups. No significant differences were observed between the groups with respect to age, gender, or baseline academic performance in Gynecology (*p* > 0.05; Table 1).

**Table 1:** Baseline characteristics of students.

Characteristics	Experimental group (*n* = 45)	Control group (*n* = 45)	*P* values
Age (median, years)	21.84 ± 0.67	21.82 ± 0.77	0.300
Gender			0.647
Male	28	26	
Female	17	19	
Baseline academic scores	82.72 ± 4.00	82.57 ± 3.42	0.369

### Comparison of teaching effectiveness

There was no statistically significant difference in pre-class test scores between the BOPPPS-CBL group and the LBL group (*p* = 0.497; Table 2). Following the teaching intervention, post-class test scores in the BOPPPS-CBL group were significantly higher than those in the LBL group (*p* < 0.05; Table 2).

**Table 2:** Test scores before and after class of two groups.

Characteristics	Experimental group (*n* = 45)	Control group (*n* = 45)	*P* values
Pre-class test scores	57.76 ± 2.68	58.24 ± 2.64	0.497
Post-class test scores	81.41 ± 3.37	70.64 ± 2.68	<0.001

### Mini-CEX and DOPS scores

The Mini-CEX comprised seven dimensions and provided a comprehensive evaluation of clinical competence. Across all dimensions, the BOPPPS-CBL group performed significantly better than the LBL group (all *p* < 0.05; Table 3). DOPS was used to assess clinical procedural skills. With the exception of the informed consent component, for which no statistically significant difference was observed between the two groups (*p* = 0.458; Table 3), all other DOPS dimensions showed statistically significant differences in favour of the BOPPPS-CBL group (*p* < 0.05; Table 3).

**Table 3:** Mini-CEX and DOPS scores of two groups.

Characteristics	Experimental group (*n* = 45)			Control group (*n* = 45)			*P* values
	Superior	Satisfactory	Unsatisfactory	Superior	Satisfactory	Unsatisfactory	
Mini-CEX							
History taking	34 (75.6%)	11 (24.4%)	0	15 (33.3%)	30 (66.75)	0	<0.001
Physical examination	36 (80.0%)	9 (20.0%)	0	16 (35.6%)	28 (62.2%)	1 (2.2%)	<0.001
Communication skills	36 (80.0%)	9 (20.0%)	0	17 (37.8%)	26 (57.8%)	2 (4.4%)	<0.001
Clinical judgment	40 (80.9%)	5 (11.1%)	0	26 (57.8%)	19 (42.2%)	0	0.001
Organization efficiency	38 (84.4%)	7 (15.6%)	0	19 (42.2%)	24 (53.3%)	2 (4.4%)	<0.001
Professionalism	44 (97.8%)	1 (2.2%)	0	36 (80.0%)	6 (13.3%)	3 (6.7%)	0.022
Overall clinical competence	38 (84.4%)	7 (15.6%)	0	22 (48.9%)	21 (31.1%)	2 (4.4%)	0.001
DOPS							
Indications and contraindications	39 (86.7%)	6 (13.3%)	0	28 (62.2%)	17 (37.8%)	0	0.016
Obtaining informed consent	41 (91.1%)	3 (6.7%)	1 (2.2%)	37 (82.2%)	5 (11.1%)	3 (6.7%)	0.458
Preparation of pre-procedure	41 (91.1%)	2 (4.4%)	2 (4.4%)	30 (66.75)	11 (24.4%)	4 (8.9%)	0.009
Technical ability	39 (86.7%)	5 (11.1%)	1 (2.2%)	26 (57.8%)	17 (37.8%)	2 (4.4%)	0.005
Communication skills	44 (97.8%)	1 (2.2%)	0	37 (82.2%)	6 (13.3%)	2 (4.4%)	0.034
Humanistic qualities/professionalism	43 (95.6%)	2 (4.4%)	0	34 (75.6%)	8 (17.8%)	3 (6.7%)	0.022
Overall ability to perform the procedure	43 (95.6%)	2 (4.4%)	0	33 (73.3%)	9 (20.0%)	3 (6.7%)	0.011

### Comparison of questionnaire results

Each questionnaire item was rated using a 5-point Likert scale. Scores of 4–5 were combined as “agreement,” a score of 3 was classified as “neutrality,” and scores of 1–2 were combined as “disagreement.” The results indicated that both teaching quality and students’ self-evaluated learning effectiveness were significantly higher in the BOPPPS-CBL group than in the LBL group (all *p* < 0.001; Table 4). However, the perceived study load was also significantly higher among students in the BOPPPS-CBL group (*p* < 0.001; Table 4).

**Table 4:** Questionnaire results of the two groups.

Characteristics	Experimental group (*n* = 45)			Control group (*n* = 45)			*P* values
	Agree	Neutral	Disagree	Agree	Neutral	Disagree	
Self-directed learning enthusiasm	39 (86.7%)	6 (13.3%)	0	11 (24.4%)	28 (62.2%)	6 (13.3%)	<0.001
Increased study workload	43 (95.6%)	2 (4.4%)	2 (4.4%)	6 (13.3%)	28 (62.2%)	11 (24.4%)	<0.001
Understanding of teaching contents	41 (91.1%)	2 (4.4%)	2 (4.4%)	15 (33.3%)	25 (55.6%)	5 (11.1%)	<0.001
Student-teacher interaction	43 (95.6%)	1 (2.2%)	1 (2.2%)	10 (22.2%)	29 (64.4%)	6 (13.3%)	<0.001
Satisfaction with teaching mode	42 (93.3%)	2 (4.4%)	1 (2.2%)	18 (40.0%)	23 (51.1%)	4 (8.9%)	<0.001
Satisfaction with teaching effectiveness	42 (93.3%)	2 (4.4%)	1 (2.2%)	11 (24.4%)	30 (66.7%)	4 (8.9%)	<0.001
Self-confidence	44 (97.8%)	0	1 (2.2%)	25 (55.6%)	16 (35.6%)	4 (8.9%)	<0.001
Interest in continuing to learn about gynaecology	45 (100.0%)	0	0	20 (44.4%)	20 (44.4%)	5 (11.1%)	<0.001

## Discussion

This research demonstrates that the BOPPPS-CBL integrated teaching model, as an innovative and effective teaching approach, has shown superior teaching effects compared with the traditional LBL teaching model in gynecological clinical internships. Specifically, it has significantly enhanced gynecological interns’ theoretical examination performance and clinical reasoning ability as evaluated by Mini-CEX. In terms of clinical skills assessed by DOPS, the model has improved most aspects of interns’ performance. Additionally, questionnaire surveys indicated that the BOPPPS-CBL model has effectively improved the teaching quality and interns’ self-evaluated learning outcomes.

In traditional gynecological internship teaching, LBL has been the predominant approach, with an emphasis on syllabus content and theoretical concepts, requiring students to passively receive information and often leading to reduced learning initiative. Consequently, this conventional teaching model no longer meets the demands of contemporary medical education. CBL enables trainees to engage in active learning within an environment closely resembling real clinical practice by introducing authentic cases and facilitating contextual analysis (17). During case discussions, trainees not only review theoretical knowledge but also integrate clinical manifestations, laboratory and imaging findings, and treatment responses to conduct systematic analysis and reasoning. This process enhances clinical thinking and problem-solving abilities (18). The BOPPPS teaching model originated in Canada and integrates Eastern and Western educational philosophies, transforming traditional didactic teaching into a participatory and heuristic approach (19). Moreover, it redefines the roles of teachers and students, emphasising student-centred learning, with teachers acting as facilitators and guides (20). Under this model, students engage in autonomous learning before, during, and after class, thereby fostering effective learning habits.

In the present study, the BOPPPS-CBL teaching model was applied to gynecological internship teaching. It should be clarified that the participants in this study were interns who were exposed to authentic clinical settings with real patients. However, gynecological internships, strict privacy requirements and patient reluctance to undergo repeated examinations often limit the amount of direct patient contact and hands-on practice available to each intern. The BOPPPS-CBL model was therefore introduced to alleviate this constraint by strengthening case analysis, clinical reasoning, and simulated doctor–patient communication training, thereby supplementing actual patient exposure while maintaining the authenticity of clinical learning. Each session was introduced using a representative clinical case, accompanied by thought-provoking questions designed to stimulate students’ interest and curiosity. Participatory learning through group discussions encouraged active engagement throughout the class, enabling students to assume a central role in the learning process and experience the benefits of deep participation. In addition, simulated doctor-patient communication scenarios not only enhanced students’ expressive abilities and adaptability but also encouraged them to consider clinical issues from the patients’ perspective, thereby strengthening doctor–patient communication skills and reinforcing a patient-centred approach to future clinical practice. Through pre-class tests, post-class tests, and summary sessions, teachers further reinforced knowledge consolidation and identified learning gaps, leading to significant improvements in learning outcomes. Overall, the teaching process was coherent and well integrated, providing a solid foundation for the sustained development of students’ learning effectiveness. In addition, it improved interns’ learning experience, learning outcomes, and satisfaction. These findings are consistent with previous studies (11, 21). Notably, all students in the experimental group reported increased interest in gynecology and expressed willingness to pursue further learning in this field, providing a strong motivational basis for the cultivation of future gynecological professionals. Therefore,although the effectiveness of CBL or BOPPPS has been previously confirmed, this study provides novel evidence by applying a structured BOPPPS-CBL integrated model in gynecological internships, where real patient exposure is limited by privacy concerns. Furthermore, our study included comprehensive outcomes including clinical skills, communication ability, and learning satisfaction, rather than only knowledge retention. These findings therefore extend previous observations and support the specific application of combined teaching models in constrained clinical environments. Nevertheless, several issues warrant consideration. On the one hand, the BOPPPS-CBL model requires instructors to possess not only solid subject expertise but also strong instructional design skills and the ability to select appropriate clinical cases. As such, this teaching approach presents new challenges for clinical teachers and necessitates continuous enhancement of teaching competence (22). On the other hand, students in the experimental group reported higher learning pressure, possibly because the model required greater engagement in pre-class preparation, in-class discussions, and post-class summarisation through mind mapping. Consequently, students perceived an increased academic burden during an already demanding internship period and may have required time to adapt to the new teaching approach.

However, with ongoing refinement and optimisation of the model, students’ learning experience is expected to improve. Furthermore, no significant difference was observed between the BOPPPS-CBL and LBL groups in obtaining informed consent. This may be attributed to the use of a relatively simple gynecological bimanual examination, which involved limited informed consent content and did not require a signed consent form. Future studies should therefore examine more complex clinical procedures with more comprehensive informed consent requirements to better evaluate the impact of different teaching models.

This study has several limitations. The sample size was relatively small, and only short-term learning outcomes of the BOPPPS-CBL model were assessed, without evaluation of its long-term effects. Future research should involve larger sample sizes, longer follow-up periods, and multicentre designs to provide more robust evidence.

## Conclusion

This study represents the first application of the BOPPPS-CBL model in gynecological clinical internship teaching. The findings indicate that this innovative teaching approach can significantly enhance interns’ theoretical performance, comprehensive clinical competence, and practical skills proficiency, while improving their learning experience and satisfaction. The BOPPPS-CBL model is therefore an effective and promising teaching strategy for gynecological clinical internships.

## Data Availability

The original contributions presented in the study are included in the article/supplementary material, further inquiries can be directed to the corresponding author/s.
